# Photodegradation of Antibiotics by Noncovalent Porphyrin-Functionalized TiO_2_ in Water for the Bacterial Antibiotic Resistance Risk Management

**DOI:** 10.3390/ijms21113775

**Published:** 2020-05-27

**Authors:** Massimiliano Gaeta, Giuseppe Sanfilippo, Aurore Fraix, Giuseppe Sortino, Matteo Barcellona, Gea Oliveri Conti, Maria Elena Fragalà, Margherita Ferrante, Roberto Purrello, Alessandro D’Urso

**Affiliations:** 1 Dipartimento di Scienze Chimiche, Università degli Studi di Catania, Viale Andrea Doria, 6 95125 Catania, Italy; gaetamassimiliano@libero.it (M.G.); giuseppe.gs416@gmail.com (G.S.); joseph.sortino@gmail.com (G.S.); mb.barcellona@gmail.com (M.B.); me.fragala@unict.it (M.E.F.); rpurrello@unict.it (R.P.); 2 Laboratory of Photochemistry, Dipartimento di Scienze del Farmaco, Università degli Studi di Catania, Viale Andrea Doria, 6 95125 Catania, Italy; fraix@unict.it; 3 Environmental and Food Hygiene Laboratory (LIAA), Department of Medical, Surgery Sciences and Advanced Technologies “G.F. Ingrassia”, University of Catania, Via Santa Sofia 87, 95123 Catania, Italy; olivericonti@unict.it (G.O.C.); marfer@unict.it (M.F.)

**Keywords:** TiO_2_, porphyrin, antibiotic, photocatalysis, noncovalent functionalization, risk management

## Abstract

Antibiotics represent essential drugs to contrast the insurgence of bacterial infections in humans and animals. Their extensive use in livestock farming, including aquaculture, has improved production performances and food safety. However, their overuse can implicate a risk of water pollution and related antimicrobial resistance. Consequently, innovative strategies for successfully removing antibiotic contaminants have to be advanced to protect human health. Among them, photodegradation TiO_2_-driven under solar irradiation appears not only as a promising method, but also a sustainable pathway. Hence, we evaluated several composite TiO_2_ powders with H_2_TCPP, CuTCPP, ZnTCPP, and SnT4 porphyrin for this scope in order to explore the effect of porphyrins sensitization on titanium dioxide. The synthesis was realized through a fully non-covalent functionalization in water at room conditions. The efficacy of obtained composite materials was also tested in photodegrading oxolinic acid and oxytetracycline in aqueous solution at micromolar concentrations. Under simulated solar irradiation, TiO_2_ functionalized with CuTCPP has shown encouraging results in the removal of oxytetracycline from water, by opening the way as new approaches to struggle against antibiotic’s pollution and, finally, to represent a new valuable tool of public health.

## 1. Introduction

Antibiotics constitute a large class of antibacterial compounds that are able to restrain or suppress the growth of microbes and pathogens [1]. Numerous drugs are natural products, or, as well, realized as synthetic and semi-synthetic chemicals [2,3,4,5]. Their classification is based on the action’s mechanism on microorganisms, spectrum activity or molecular structure [1,6,7]. Antibiotics have revolutionized the medical sciences and our modern way of living due to their capabilities to prevent and tackle human infectious diseases [8,9,10,11], representing a landmark of public health in the reduction of risk by infection diseases. In addition to human treatments, they have also been employed in veterinary medicine [12,13] and livestock farming [11,14].

The antibiotic usage represents a still current risk for its impact on the environmental pollution in water and, finally, in food quality, although this aspect is managed already through dedicated policies in European community [11,15,16,17,18]. The global antibiotic consumption is estimated to exceed 100,000 tons per year, in a rapid growth which will bring by another 67% only in animal husbandry by 2030 including aquaculture [19,20].

The environmental regulation on a local and/or global scale is inadequate to fully manage this problem, in fact, urban, hospital, veterinary, and pharmaceutical industry wastewaters, illegal drug disposals, and aquaculture can represent significant sources of underestimated pollution in freshwater, seawater, soil, and all aquatic ecosystems [11,20]. Moreover, the disposed wastewaters contain antibiotics, due to the lack of specific legal standard limits that are aimed at the management of wastewater treatment plants (WWTPs) [21]. As a consequence, even tap water is susceptible to antibiotics’ contamination, ranging from few to hundreds of nanograms per litre in different countries [11,22,23]. Although no direct effects between human health and traces of antibiotics in drinking water have been reported [24,25], it is well-established that sub-lethal concentrations can lead to the antibiotic resistance [26,27,28,29], representing, then, an emerging problem of public health.

Bacteria can develop resistance due to intrinsic mechanisms that can be resistant (for example, Gram-negative bacteria are not susceptible to glycopeptides) or ex-novo acquired through resistance gene transmission that is achieved by mutations in different chromosomal loci or horizontal acquisition of resistance genes (by plasmids, integrons, or transposons), with the greatest concern placed on the bacteria that have acquired transferable antibiotic resistance determinants [30]. Regarding the development of antibiotic-resistant bacteria through the plasmidic route, the plasmid can remain both integrated in the cellular DNA or as free state in the cytoplasm. Some conjugative plasmids possess a set of genes devoted to promote their horizontal transmission to different cells. More plasmids can accumulate a form of multiple resistance that thwarts the effect of several antibiotics, rendering any antibacterial therapy ineffective. Antibiotic-resistant bacteria each year provoke 25,000 deads in European population and almost half a million all over the world, due to relative intractable bacterial infections [31,32] increasing also economic and health burdens of antibiotic resistance diseases. In the USA alone, the national annual cost for managing bacteria-resistant diseases exceeds $2.2 billion, based on the number of cases in 2014 [33].

As a result, it is highly recommended to develop appropriate and innovative strategies and/or methodologies to remove antibiotics from contaminated waters. In industries, the adsorption method that is by activated carbon is widely used for eliminating organic pollutants by wastewaters, antibiotics included [11,34,35,36]. In alternative to activated carbon, magnetic nanoparticles based on iron particles and nanosized modified carbon particles have been reported, so as to facilitate the recycle and separation process for the adsorbent materials [36,37]. Other techniques that are applied in water purification include nanofiltration on membrane [38], ozonation [39], and photo-Fenton oxidation [40]. However, most of the existing adsorption techniques are not able to eliminate all of the antibiotic load in water, and successive methods have to be employed to maximize their removal [41]. In this respect, photodegradation and advanced oxidation processes appear as intriguing tools to finally achieve optimal treatment of water [42,43].

Among them, titanium dioxide (TiO_2_) is a promising photocatalytic semiconductor that is involved in technological wastewater treatment by UV-A or simple solar irradiation use [44]. Commercial availability, low-cost, absence of toxicity, and photochemical stability, make TiO_2_ a practically-ideal photocatalyst in the treatments of water [45,46]. The photocatalytic process starts when photons, having an energy higher than TiO_2_ bandgap, excite electrons from the valence band to the conduction band. As a consequence, the vacancies in the valance band can oxidize the water molecules or hydroxyl ions adsorbed onto TiO_2_ surface generating hydroxyl radicals [46,47]. Hydroxyl radicals (OH∙) are strong oxidant and they are responsible for the degradation of pollutants.

Oxolinic acid (OXA) and oxytetracycline (OTC) antibiotics are two of the most extensive antibiotics that are employed in aquaculture [48,49]. Suspensions of TiO_2_ have been successfully used to degrade OXA and OTC in water under solar irradiation [50,51,52,53]. However, TiO_2_ is a wide bandgap semiconductor that can only harvest UV light, limiting the photo-response under visible-light [54]. In light of these considerations, the photosensitization process of wide band-gap semiconductors can improve their efficiency under visible solar irradiation [55].

Porphyrins have been reported as common photosensitizers for TiO_2_ [56] and for the generation of singlet oxygen (^1^O_2_) due to their strong absorption in the visible region (400–450 nm) and huge molar extinction coefficient [57,58,59].

Especially, 5,10,15,20-tetrakis(4-carboxyphenyl)-porphyrin (H_2_TCPP) and its zinc(II) derivative (ZnTCPP) enhances the quantum efficiency of TiO_2_ [60,61]. Porphyrin modified-TiO_2_ has been synthesized via either chemical functionalization or mixed covalent/noncovalent approach, in organic solvents and in water, for *i)* photocatalytic degradation of organic pollutants [62,63,64,65] and *ii)* water sanification and antimicrobial applications [66,67,68].

**Figure 1:**
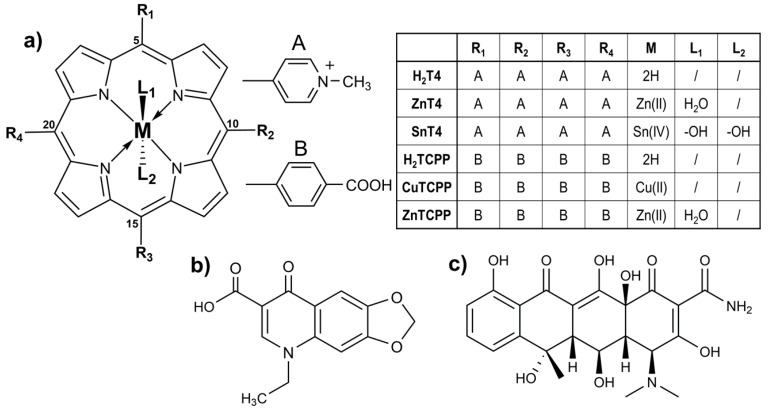
Molecular structures of (**a**) porphyrins used, (**b**) oxolinic acid, and (**c**) oxytetracycline.

However, porphyrin functionalized TiO_2_ nanomaterials have not been investigated yet to photodegrade antibiotics in aqueous solution. In this work, an environmental-friendly synthesis of porphyrin@TiO_2_ has been achieved by a non-covalent one-pot approach in water at room conditions. Sn(IV) 5,10,15,20-tetrakis(4-pyridyl)porphyrin (SnT4, Chart 1a), H_2_TCPP (Figure 1a), and its copper (II) and zinc (II) derivatives (CuTCPP, ZnTCPP. Chart 1a), have been used to functionalize TiO_2_ nanoparticles in pure water. Moreover, TiO_2_, alone and the final composites, have been employed to test the photodegradation process of both two antibiotics, OXA (Figure 1b) and OTC (Figure 1c) in neutral water under simulated solar irradiation for evaluating, finally, this methodology as tool of public health in water and wastewaters management, including waters of acquaculture plants. Porphyrin@TiO_2_ composite materials have shown encouraging results that pave the way a novel and sustainable approach for removing antibiotic pollutants from contaminated water.

## 2. Results and Discussions

### 2.1. Selection of Porphyrinoid Systems

Porphyrinoids have been demonstrated to be active producer of ^1^O_2_ as much as effective photosensitizers for TiO_2_. In the interest to select efficient porphyrin systems, production of ^1^O_2_ in deuterated water was first evaluated. Although the behaviour of the porphyrin alone in water might be different when compared to that on the surface, the characterizations in aqueous solution can offer a valuable guide to extend the investigations on functionalized systems and rationalize the findings.

The screening of porphyrins properties was carried out among tetra-anionic (H_2_TCPP, CuTCPP, ZnTCPP) and tetra-cationic (H_2_T4, SnT4, ZnT4) systems, by measuring the typical phosphorescence of ^1^O_2_ in the near-IR (ca. 1270 nm), upon excitation with 405 nm laser light (Figure S1). Comparable productions of ^1^O_2_ are observed in the presence of cationic porphyrins, H_2_T4, SnT4, and ZnT4 (Figure S1a). Conversely, in anionic systems, the highest yields of ^1^O_2_ are revealed in the presence of H_2_TCPP, whereas the ZnTCPP sample exhibits a moderate activity (Figure S1b). It is worth noting that the lower efficiency of ZnTCPP can be explained by a massive photodegradation as confirmed by the drastic decrease of the absorption spectrum (data not shown). As expected under these conditions, CuTCPP does not generate ^1^O_2_ upon irradiation, owing to the negligible emission provided, which is ascribed to the geometry of Cu porphyrins (Figure S1b, red dots).

In view of foregoing, we have decided to functionalize the TiO_2_ surface with all tested tetra-carboxylate porphyrins, which can also give a further binding as a consequence of H-bond formation with the TiO_2_ surface [67]. Noteworthy, even if CuTCPP was found to not be able to generate ^1^O_2_, we tested it in order to study the central metal effect.

SnT4 was mainly chosen due to its two hydroxyls as axial ligands in water, which can provide additional H-bond with the TiO_2_ surface, and for its high tendency to form O-bound complexes, despite the similar evidence in cationic systems, SnT4 [69].

### 2.2. Functionalization of TiO_2_

**Figure 2:**
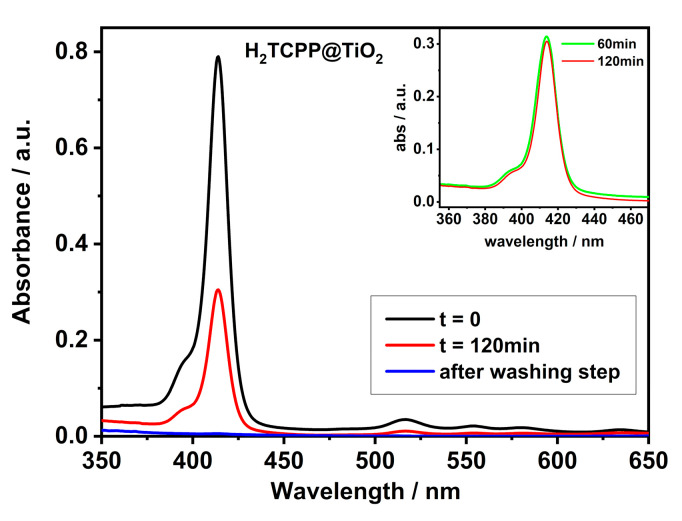
UV/Vis spectra (cuvette path length = 0.1 cm) of H_2_TCPP aqueous solution (20 µM, pH = 5.8, black curve) and after 120 min. in the presence of 20 mg TiO_2_ (red curve). The blue curve refers to the absorption of the supernatant solution after the H_2_TCPP@TiO_2_ powder’s washing step. Inset: comparison between the absorbance of the same solution after 60 min. (green curve) and 120 min. (red curve) of functionalization.

Water solution (20 µM) of tetracarboxylate free-base porphyrin (H_2_TCPP) at pH = 5.8 shows an intense Soret band that is centred at 414 nm and four Q-band at 516 nm, 552 nm, 591 nm, and 645 nm, (Figure 2). We have added 20 mg of TiO_2_ to the solution of porphyrin and they have left the suspension 2 h under vigorous stirring. Noteworthy, a significant reduction (~60%) of the porphyrin Soret band intensity has been detected after separation of the solid by centrifugation (Figure 2). These findings can be attributed to the dye-adsorption onto the titanium dioxide’s surface, since we observed an intense coloured powder as final product. No porphyrin desorption from TiO_2_ surface is experienced during precipitate washing, as demonstrated by the UV-spectrum of the resulting supernatant solution (Figure 2, blue line). Noteworthy, the adsorption is already almost completed in one hour from the start (Figure 2-inset), so that 2 h can represent an optimal time for functionalizing the TiO_2_ system in of these conditions.

The point of zero charge (pzc) is an important surface parameter, which is used extensively in characterizing the net surface charge of an adsorbent in aqueous phase, in order to exploit the electrostatic interactions as driving force to functionalize the TiO_2_ surface. More in detail, when the pH of the bulk solution is equal to the pH_pzc_ of the nanomaterials, the surface potential will be zero. As a consequence, at pH values above pH_pzc_, the nanomaterial’s surface will assume a negative charge, more efficiently interacting with positively charged molecules [70,71]. The pH_pzc_ of TiO_2_ (P25 from Degussa) is 6.2, as reported previously [71,72]. At the same time, even the pKa of the porphyrin moieties have been considered: the protonation of the pyrrole nitrogens in H_2_TCPP has been reported to occur at pH below 5, and the pKa of the carboxylic acid moieties is also approximately 5 [73,74]. Therefore, in our experimental conditions (pH = 5.8), H_2_TCPP porphyrin presents anionic charge due to the carboxylic groups partially deprotonated (even if a small percent of protonated core can not be excluded), and TiO_2_ surface slightly positive. Consequently, the coulombic interactions are the main driving forces that encourage the porphyrins to interact with the titanium dioxide. Moreover, we can suppose the formation of hydrogen bonds between protonated carboxylic groups and terminal hydroxyl groups (-OH) onto the TiO_2_ surface. The network of interactions stabilizes the structure and makes the functionalization strong enough to avoid release of porphyrin in water, even after the solid washing step (Figure 2).

Similar results have been obtained for the systems CuTCPP@TiO_2_ (Figure S2), and ZnTCPP@TiO_2_ (Figure S3), in which more efficient adsorptions (more than 95%) of porphyrin is detected (Table 1) as a result of stronger interactions. The presence of central metal prevents the protonation of the pyrrole nitrogens in the porphyrin core, thus minimising the repulsions with TiO_2_ surface (slightly positive).

Conversely, the functionalization with SnT4 does not appear to be extremely efficient: the adsorption rate stops at about 30% (Figure S4 and Table 1). SnT4 porphyrin is a permanent tetra-cation macrocycle, which, in strong basic conditions (pH = 12.0), is able to interact with TiO_2_ negative surface via electrostatic forces. However, the absence of relevant hydrogen bonds explains both the weak adsorption of SnT4 and release of porphyrin during the washing stage. Noteworthy, the presence of some H-bond is undeniable, but not able to guarantee an incisive anchoring onto TiO_2_ surface, owing to OH- axial ligands in SnT4.

### 2.3. Photodegradation Tests on Oxolinic Acid and Oxytetracycline

**Figure 3:**
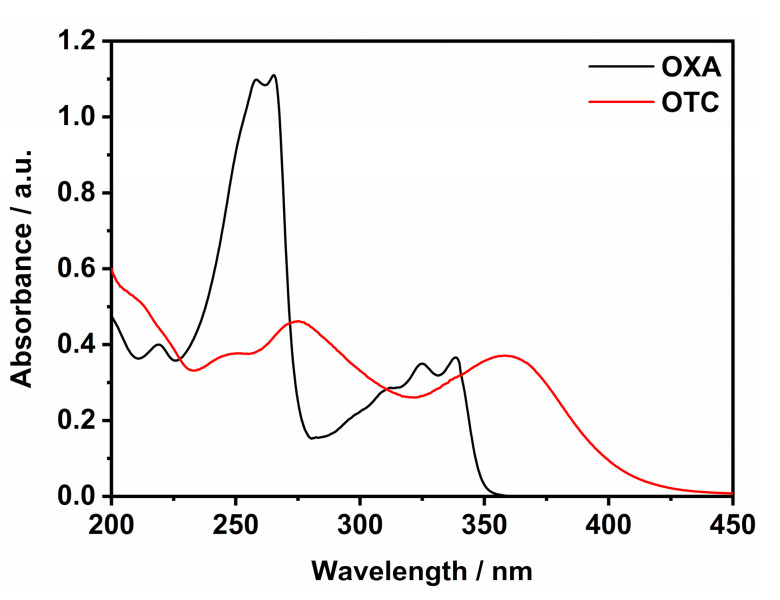
UV/Vis spectra (path length = 1 cm) of water solutions of OXA (30 µM, pH = 7.0, black curve) and OTC (30 µM, pH = 7.0, red curve).

OXA (Figure 1b) is a nonfluorinated quinolone antibiotic that is largely used in human and veterinary medicine for treating numerous infectious. In aqueous solution, the UV/Vis spectrum of OXA (Figure 3, black trace) shows different peaks, which can be gathered in two macro-range: the first one, between 200 and 300 nm, attributable to π→π* electronic transitions of aromatic rings; the second region, included between 300 and 350 nm, is due to the n→π* transitions, with HOMO-LUMO absorption being centred at about 340 nm [75]. However, OXA owns a deprotonable carboxylic group (pK_a_ = 6.92 [76]) that can shift slightly the peak around 340 nm as a function of pH values. At neutral conditions (pH = 7.0), OXA appears as partially deprotonated (almost 50%) [53], and the main peak falls on 338 nm (Figure 3, black trace) [75].

OTC (Figure 1c) is a tetracycline antibiotic that is extensively employed in agriculture and aquaculture. In water solution, OTC shows three different protonation steps (pK_a1_ = 3.22, pK_a2_ = 7.46, pK_a3_ = 8.94), depending on pH conditions [53], which can affect the absorbance properties. In fact, the UV/Vis characterization (Figure 3, red trace) evidences two broad absorption regions, 225–325 nm and 325–450 nm, where the peak around 360 nm is ascribable to HOMO-LUMO transitions as above-mentioned for OXA [53,75,77,78]. At neutral conditions (pH = 7.0), almost 50% of OTC is negatively charged, owing to the second deprotonation [53], and the main peak shifts to 359 nm (Figure 3, red trace).

**Figure 4:**
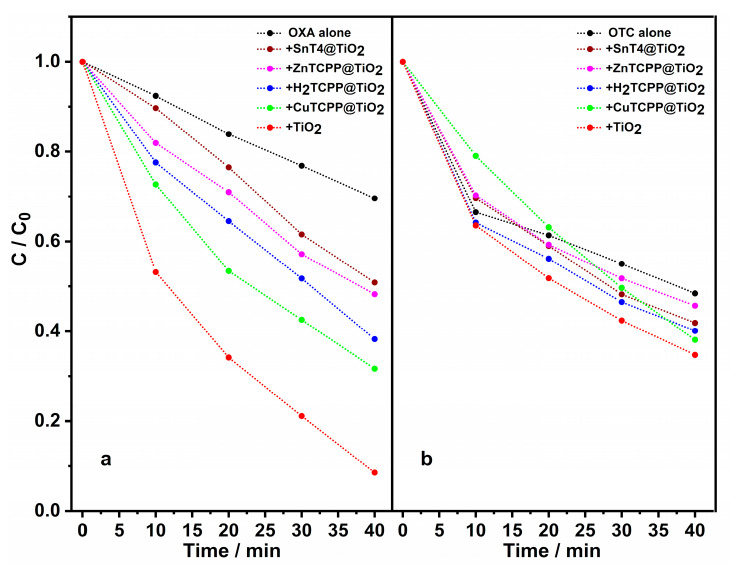
Photodegradation rates as function of C/C_0_ *vs* irradiation time for OXA (panel **a**, *λ_max_* = 338 nm) and OTC (panel **b**, *λ_max_* = 359 nm): alone (black dots) and in the presence of SnT4@TiO_2_ (wine dots), ZnTCPP@TiO_2_ (purple dots), H_2_TCPP@TiO_2_ (blue dots), CuTCPP@TiO_2_ (green dots) naked TiO_2_ (red dots). In all experiments, the antibiotics’ initial concentration was 30 µM at pH = 7.0, and the amount of photocatalyst used was 1 mg.

The irradiation of an aqueous solution of OXA (3 × 10^–5^ M, pH = 7.0) yields to photodegradation, as evidenced from the reduction of its absorbance with time (Figure S5, panel a). The introduction of TiO_2_ as photocatalyst entails a vertiginous increase of the antibiotic decomposition (Figure S5, panel b). The photodegradation rate can be better evaluated by reporting the normalized changes in concentration (C/C_0_) *vs* irradiation time (Figure 4). As expected, the degradation rate in the presence of photocatalyst TiO_2_ (Figure 4a, red dots) is faster when compared to OXA alone in solution (Figure 4a, black dots). Furthermore, composite porphyrin-TiO_2_ materials have been proved for testing their effectiveness in degrading oxolinic acid in identical conditions (Figure 4a and Figure S6). On the contrary to the preliminary investigation done in aqueous solution, SnT4@TiO_2_ shows the worst photocatalytic activity with respect to other systems (Figure 4a, wine dots and Figure S6, panel a), reasonably due to the scarce surface functionalization as evidenced in Table 1. Overall, in the remaining systems (Figure S6, panels b-c-d), an improvement of the photocatalytic properties was observed, reaching positive outcomes for CuTPPS@TiO_2_ (Figure 4a, green dots).

The effect of the simulated solar irradiation on a water solution of OTC (3 × 10^–5^ M, pH = 7.0) reveals more than 30% of photodegradation after 10 min. (Figure S7, panel a) compared to OXA. The presence of TiO_2_ does not affect the photodegradation process within 10 min.; however, the degradation rates gradually become more divergent with time (Figure 4b and Figure S7). The other porphyrin@TiO_2_ systems display analogous trends (Figure 4b and Figure S8, panels a-b-c-d), except for CuTCPP@TiO_2_ (Figure 4b, green dots). In this latter case, the photodegradation rate proceeds linearly with time up to a comparable C/C_0_ value to that of TiO_2_ alone. By comparing the evolution of the photocatalytic activity of CuTCPP@TiO_2_ and titanium dioxide alone, one might speculate that, beyond 40 min. of irradiation, CuTCPP@TiO_2_ seems to be more efficient than TiO_2_.

The mechanism that is involved in the contaminant degradation in presence of the modified TiO_2_ remains to be investigated further, especially the contribution of the porphyrin in the photogenerated electron donation and/or photogeneration of ^1^O_2_. Nevertheless, CuTCPP has already shown distinct photoproperties with respect to the other tested porphyrins during the preliminary investigation in solution and it seems to be an excellent outset to go further in the comprehension of OTC degradation.

## 3. Materials and Methods

Oxolinic acid (OXA, MW = 261.2, CAS number 14698-29-4) and oxytetracycline hydrochloride (OTC, MW = 496.89, CAS number 2058-46-0) were purchased from Sigma–Aldrich Company and used without further purification. Stock solutions (both 7 × 10^–3^ M, pH = 12 by NaOH 6M) of OXA and OTC were prepared freshly before use, by dissolving an exact amount of the solid in ultrapure water obtained from Elga Veolia PURELAB flex and stored in the dark at 4 °C.

H_2_TCPP, CuTCPP, ZnTCPP, H_2_T4, ZnT4, and SnT4 porphyrin were purchased from Sigma–Aldrich Company and used without further purification. Porphyrin stock solutions (about 4 × 10^–4^ M) were prepared in ultrapure water obtained from Elga Veolia PURELAB flex and stored in the dark at room temperature. The concentrations of these solutions were spectrophotometrically calculated (UV/Vis in H_2_O at neutral pH) by monitoring the maximum intensity of the respective Soret bands: λ_max_(H_2_O) = 414 nm (ε = 386,000 dm^3^ mol^−1^ cm^−1^) for H_2_TCPP; λ_max_(H_2_O) = 414 nm (ε = 364,000 dm^3^ mol^−1^ cm^−1^) for CuTCPP; λ_max_(H_2_O) = 421.5 nm (ε = 376,000 dm^3^ mol^−1^ cm^−1^) for ZnTCPP; λ_max_(H_2_O) = 423 nm (ε = 226,000 dm^3^ mol^−1^ cm^−1^) for H_2_T4; λ_max_(H_2_O) = 437 nm (ε = 204,000 dm^3^ mol^−1^ cm^−1^) for ZnT4; λ_max_(H_2_O) = 422 nm (ε = 215,000 dm^3^ mol^−1^ cm^−1^) for SnT4.

Titanium dioxide (TiO_2_, named P25, ca. 70% anatase and 30% rutile, size ≈ 20–30 nm, surface area 50 ± 15 m^2^/g) was purchased from Degussa Company.

Uv/Vis measurements were carried out at room temperature (298 K) on a JASCO V-530 spectrophotometer.

### 3.1. Measurements of ^1^O_2_ in Water Solution

The solutions were prepared in D_2_O with 5% on H_2_O, so that to be optically matched at 405 nm in order to evaluate the production of ^1^O_2_ generated by H_2_TCPP, CuTCPP, ZnTCPP, H_2_T4, ZnT4 and SnT4 alone in water. ^1^O_2_ emission was registered with a Fluorolog-2 (Model, F111) spectrofluorimeter equipped with a NIR-sensitive liquid nitrogen cooled photomultiplier irradiating air saturated samples at 405 nm a laser (50 mW). The measurements were performed at room temperature (298 K) and a quartz cuvette (path length = 1 cm) was used.

### 3.2. Preparation of Porphyrin@TiO_2_ Nanomaterials

20 mg of TiO_2_ were added to 5 mL of porphyrin (H_2_TCPP@TiO_2_, CuTCPP@TiO_2_, and ZnTCPP@TiO_2_) water solution (20 µM) at pH = 5.8, adjusted with HCl 1M. For SnT4@TiO_2_ same amounts and procedures were used, except for pH, which was fixed to 12.0 with NaOH 6M for the entire functionalization time. The suspension was stirred in the dark at room temperature for 2 h. The solids were isolated by centrifugation (30 min, 5000 rpm). The solution has been analyzed by UV-Vis spectroscopy in order to determine the remaining amount of porphyrins in solution and, hence, for difference, the amount of porphyrin deposited onto TiO_2_ surface. After separation, the powders were washed with ultrapure water and dried in the air for 48 h. Direct UV-Vis analysis of the solution resulting from the washing process without further dilution have been performed in order to estimate the amount of porphyrin loaded on the TiO_2_ surface and the release of porphyrin in solution. For these experiments, a quartz cuvette with 0.1 cm path-length has been used. In order to avoid any interference with the spectroscopic data, owing to stick of porphyrin onto the container walls, we have performed the functionalization of TiO_2_ in plastic falcon.

### 3.3. Photocatalytic Reactions

The photoreactor consisted of a borosilicate beaker (100 mL), illuminated from the top (10 cm away from the testing solution) by Osram Ultra Vitalux 300 W E27 lamp, specially designed for sunlight simulation. The entire system, including the magnetic stirrer, is enclosed in a ventilated box. Antibiotic solutions (50 mL), containing 1 mg of TiO_2_ or porphyrin@TiO_2_ in suspension, were used as photocatalytic samples. The initial pH was adjusted at neutral conditions with HCl 6M. The initial concentration of the antibiotic solution was 30 µM for both OXA and OTC.

Before the experiments, the system was kept in darkness for 15 min. with vigorous stirring in order to reach the equilibrium adsorption in the photocatalytic system. Subsequently, the system was irradiated under stirring and, at regular time intervals, the solution was withdrawn in a quartz cuvette with 1 cm path-length, without further filtration, in order to determine the residual antibiotic by UV/Vis measurements. The degradation was evaluated by the absorbance peak at 338 nm for OXA and at 359 nm for OTC and then converted in concentration using a Beers law calibration curve. Moreover, the photodegradation rate was calculated as C/C_0_ against the irradiation time, where C is the concentration after irradiation and C_0_ is the initial concentration. Finally, photocatalytic experiments were performed in the absence of the photocatalyst, using the same experimental setup previously described.

## 4. Conclusions

The photolysis experiments indicate that solar irradiation is not so efficient to eliminate OXA as much as OTC. The reason might be found in the comparison of the UV/Vis spectra of both drugs: OTC absorbs in a broad region of the visible light, whereas OXA mainly absorbs in the ultraviolet and blue regions of the solar spectrum. It is well known that any organic contaminants are mineralized into CO_2_, water and inorganic compounds via photo-oxidation with light [79]. In particular, the photo-mineralization of OTC leads to significant amounts of ammonium, (55% of stoichiometric quantity) in comparison with OXA (40% of stoichiometric quantity) [53]. In our experimental conditions (i.e., pH = 7.0) the TiO_2_ surface is slightly negatively charged, and therefore able to attract cationic ammonium molecules. This phenomenon could respond to the reduced photodegradation rates for OTC after 10 min., even in presence of bare photocatalyst and functionalized photocatalyst. However, CuTCPP@TiO_2_ appears as a promising system and *species-specific* for the photo-removal of OTC in water.

It is worth considering that the porphyrinoids’ photosensitizing properties towards TiO_2_ could compete with their self-capability to generate singlet oxygen and reactive oxygen species, ROS. Further, the TiO_2_ photocatalyst has been reported to be a potential deactivator of ^1^O_2_ in aqueous solution [80]. As a result, this two-fold phenomena may limit the final effectiveness of our systems. However, the functionalization mode of porphyrin molecules onto photocatalyst semiconductors is an important aspect [56] that make our results significant steps to optimize the overall performance. Besides, porphyrin sensitized TiO_2_ photocatalysts constitute fascinating hybrid organic/inorganic materials having real perspectives in water purification.

Nevertheless, our porphyrin functionalized TiO_2_ nanomaterials can represent an innovative tool for depuration of waters and wastewaters by OTC contamination, aimed to improve the reduction of bacterial resistance to antibiotics and, finally, to reduce the health outcomes related to the inefficacy of current pharmacological therapies against these diseases.

## Figures and Tables

**Table 1:** Percentage of adsorption and quantification of the investigated systems.

System	% _adsorption_^1^	Quantification ^2^
H_2_TCPP@TiO_2_	≈ 60	≈ 2.5
CuTCPP@TiO_2_	≈ 95	≈ 4.0
ZnTCPP@TiO_2_	≈ 100	≈ 4.0
SnT4@TiO_2_	≈ 30	≈ 1.0

^1^ The adsorption rate is calculated respect to the initial concentration of porphyrin. ^2^ Quantification is expressed as mg_porph_/gr_TiO2_. Adsorption rate and quantification are intended as approximate estimates.

## Supplementary Materials

Supplementary materials can be found at https://www.mdpi.com/1422-0067/21/11/3775/s1.

## Abbreviations

OXA	Oxolinic acid
OTC	Oxytetracycline
H_2_T4	5,10,15,20-tetrakis(4-pyridyl)porphyrin
ZnT4	Zn(II) 5,10,15,20-tetrakis(4-pyridyl)porphyrin
SnT4	Sn(IV) 5,10,15,20-tetrakis(4-pyridyl)porphyrin
H_2_TCPP	5,10,15,20-tetrakis(4-carboxyphenyl)porphyrin
CuTCPP	Copper(II) 5,10,15,20-tetrakis(4-carboxyphenyl)porphyrin
ZnTCPP	Zinc(II) 5,10,15,20-tetrakis(4-carboxyphenyl)porphyrin
