# Supplementary material for: Photodegradation of Antibiotics by Noncovalent Porphyrin-Functionalized TiO2 in Water for the Bacterial Antibiotic Resistance Risk Management
Source: Int J Mol Sci. 2020 May 27;21(11):3775. doi: 10.3390/ijms21113775 (PMC7312883; doi:10.3390/ijms21113775)
Supplement: Supplementary file 1 [file ijms-21-03775-s001.pdf]

Supplementary materials

Photodegradation of antibiotics by noncovalent porphyrin-functionalized TiO_2 in water for the bacterial antibiotic resistance risk management

Massimiliano Gaeta [1], Giuseppe Sanfilippo [1], Aurore Fraix [2], Giuseppe Sortino [1], Matteo Barcellona [1], Gea Oliveri Conti [3], Maria Elena Fragalà [1], Margherita Ferrante [3], Roberto Purrello [1] and Alessandro D'Urso [1,*]

[1] Dipartimento di Scienze Chimiche, Università degli Studi di Catania, Viale Andrea Doria, 6 95125 Catania, Italy; gaetamassimiliano@libero.it (M.G.); giuseppe.gs416@gmail.com (G.S.1); joseph.sortino@gmail.com (G.S.2); me.fragala@unict.it (M.E.F.); rpurrello@unict.it (R.P.)

[2] Laboratory of Photochemistry, Dipartimento di Scienze del Farmaco, Università degli Studi di Catania, Viale Andrea Doria, 6 95125 Catania, Italy; fraix@unict.it (A.F.)

[3] Environmental and Food Hygiene Laboratory (LIAA), Department of Medical, Surgery Sciences and Advanced Technologies "G.F. Ingrassia", University of Catania, Via Santa Sofia 87, 95123, Catania, Italy; marfer@unict.it (M.F.); olivericonti@unict.it (G.O.C.)

* Correspondence: adurso@unict.it (A.D.)

Table of Contents

Figure S1 - $^1O_2$ emission for aqueous solutions of H_2T4/ZnT4/SnT4/H_2TCPP/CuTCPP/ZnTCPP 2

Figure S2 - UV/Vis spectra of TiO_2-functionalization with CuTCPP 3

Figure S3 - UV/Vis spectra of TiO_2-functionalization with ZnTCPP 4

Figure S4 - UV/Vis spectra of TiO_2 functionalization with SnT4 5

Figure S5 - UV/Vis photodegradation spectra of OXA alone and OXA+TiO_2 6

Figure S6 - UV/Vis photodegradation spectra of OXA + porphyrin@TiO_2 7

Figure S7 - UV/Vis photodegradation spectra of OTC alone and OTC+TiO_2 8

Figure S8- UV/Vis photodegradation spectra of OTC + porphyrin@TiO_2 9

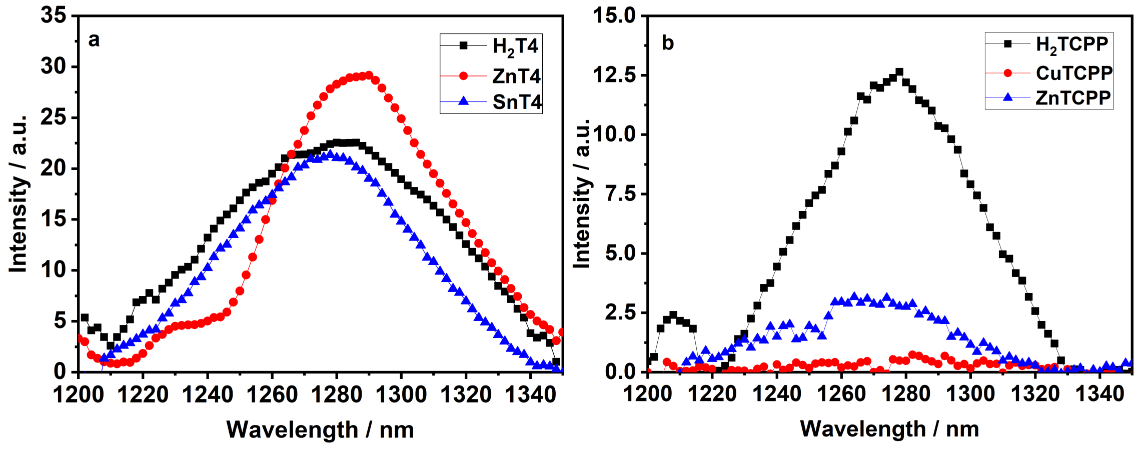

Figure S1. $^1O_2$ luminescence detected upon 405 nm light excitation of optically matched solutions of H_2T4. ZnT4, SnT4 (panel a) and H_2TCPP, CuTCPP, ZnTCPP (panel b) in D_2O with 5% on H_2O

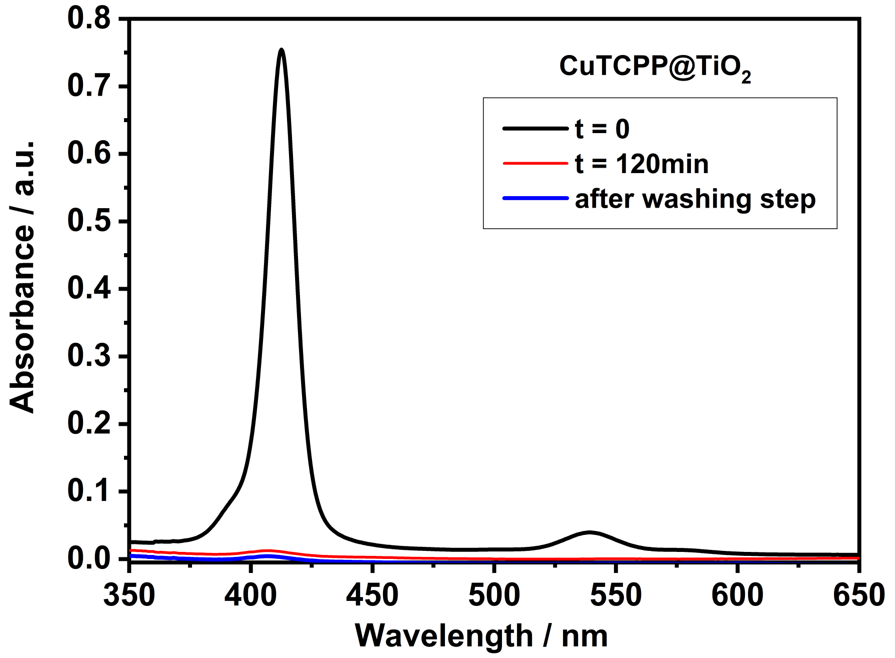

Figure S2. UV/Vis spectra (cuvette path length = 0.1 cm) of CuTCPP aqueous solution of (20 μM, pH=5.8, black curve) and after 120 minutes in the presence of 20 mg TiO_2 (red curve). The blue curve refers to the absorption of the supernatant solution after the CuTCPP@TiO_2 powder's washing step.

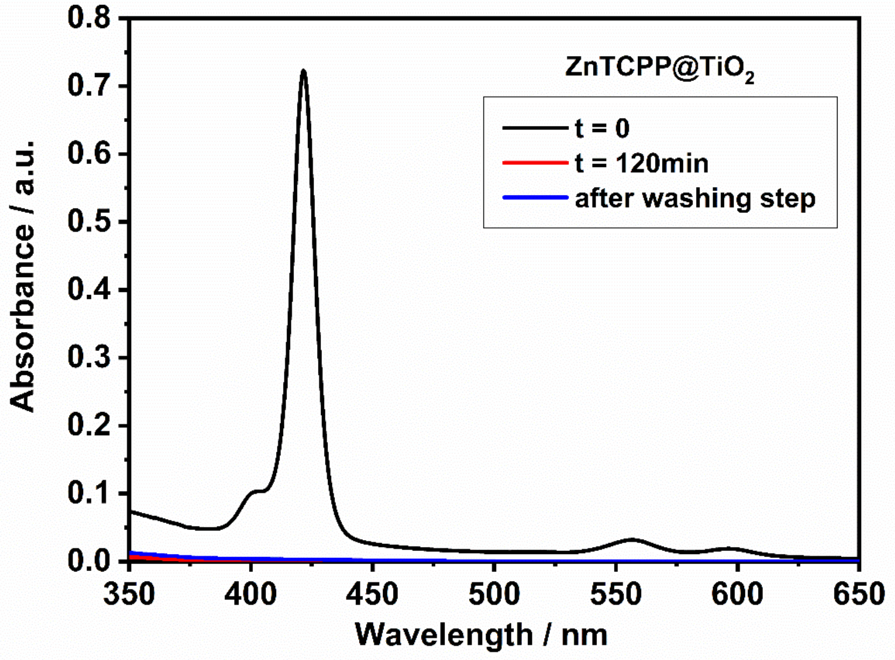

Figure S3. UV/Vis spectra (cuvette path length = 0.1 cm) of an aqueous solution of ZnTCPP at zero time (20 μM, pH=5.8, black curve) and after 120 minutes in the presence of 20 mg TiO_2 (red curve). The blue curve refers to the absorption of the supernatant solution after the ZnTCPP@TiO_2 powder's washing step.

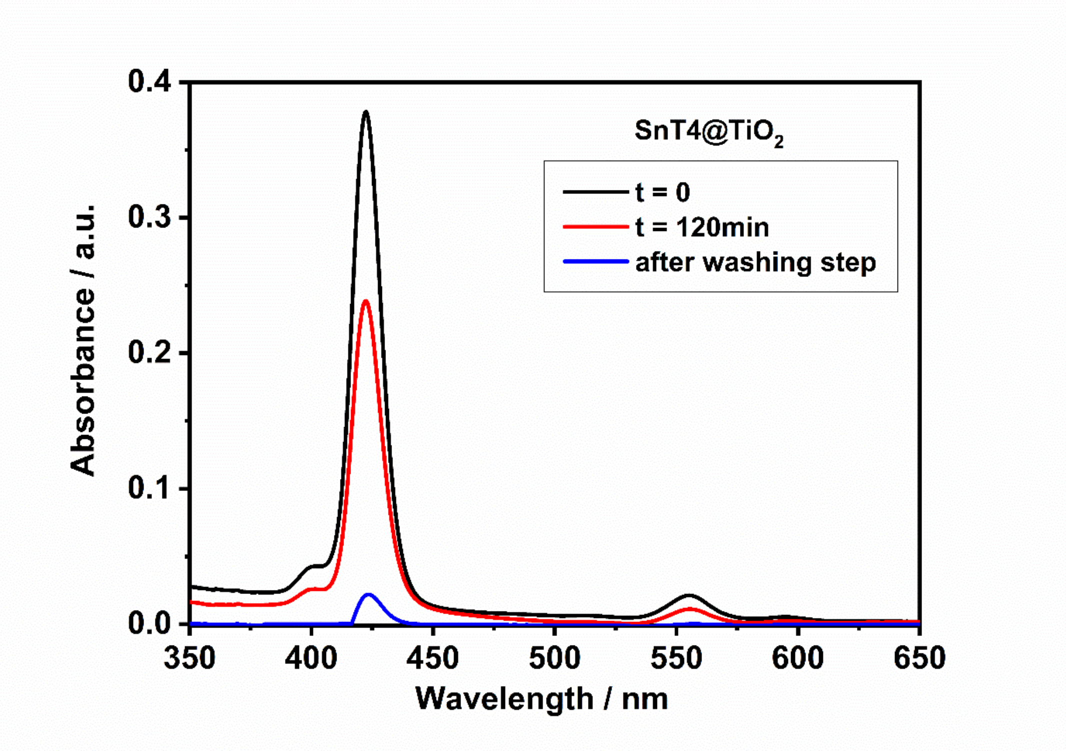

Figure S4. UV/Vis spectra (path length = 0.1 cm) of SnT4 aqueous solution (20 μM, pH = 12, black curve) and after 120 minutes in the presence of 20 mg TiO_2 (red curve). The blue curve refers to the absorption of the supernatant solution after the SnT4@TiO_2 powder's washing step.

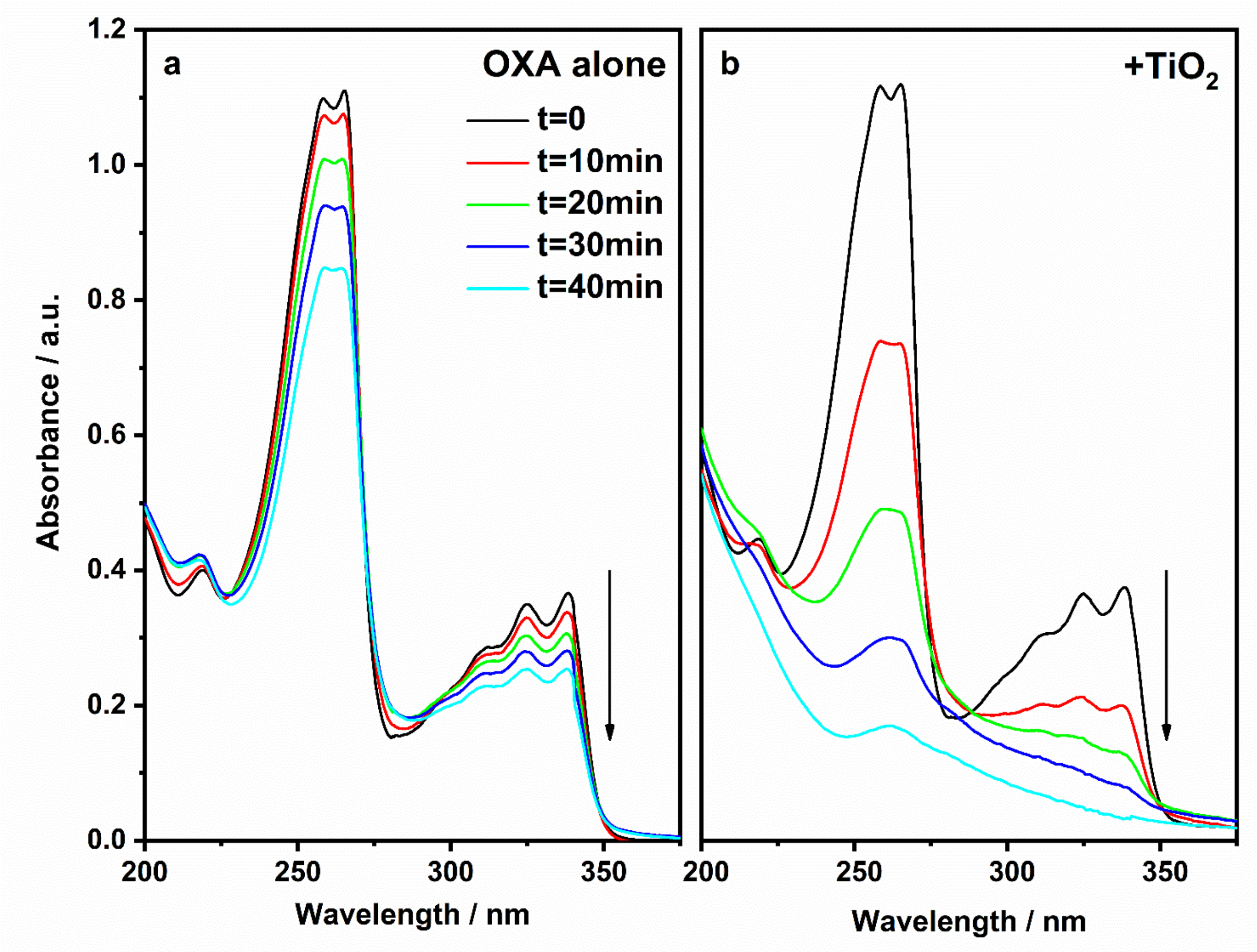

Figure S5. UV/Vis spectra (cuvette path length = 1 cm) of the photodegradation tests at different irradiation time for water solution (pH=7.0) of OXA alone (panel a) and in presence of 1 mg of TiO_2 (panel b). In both experiments, the initial concentration of OXA is 30 μM.

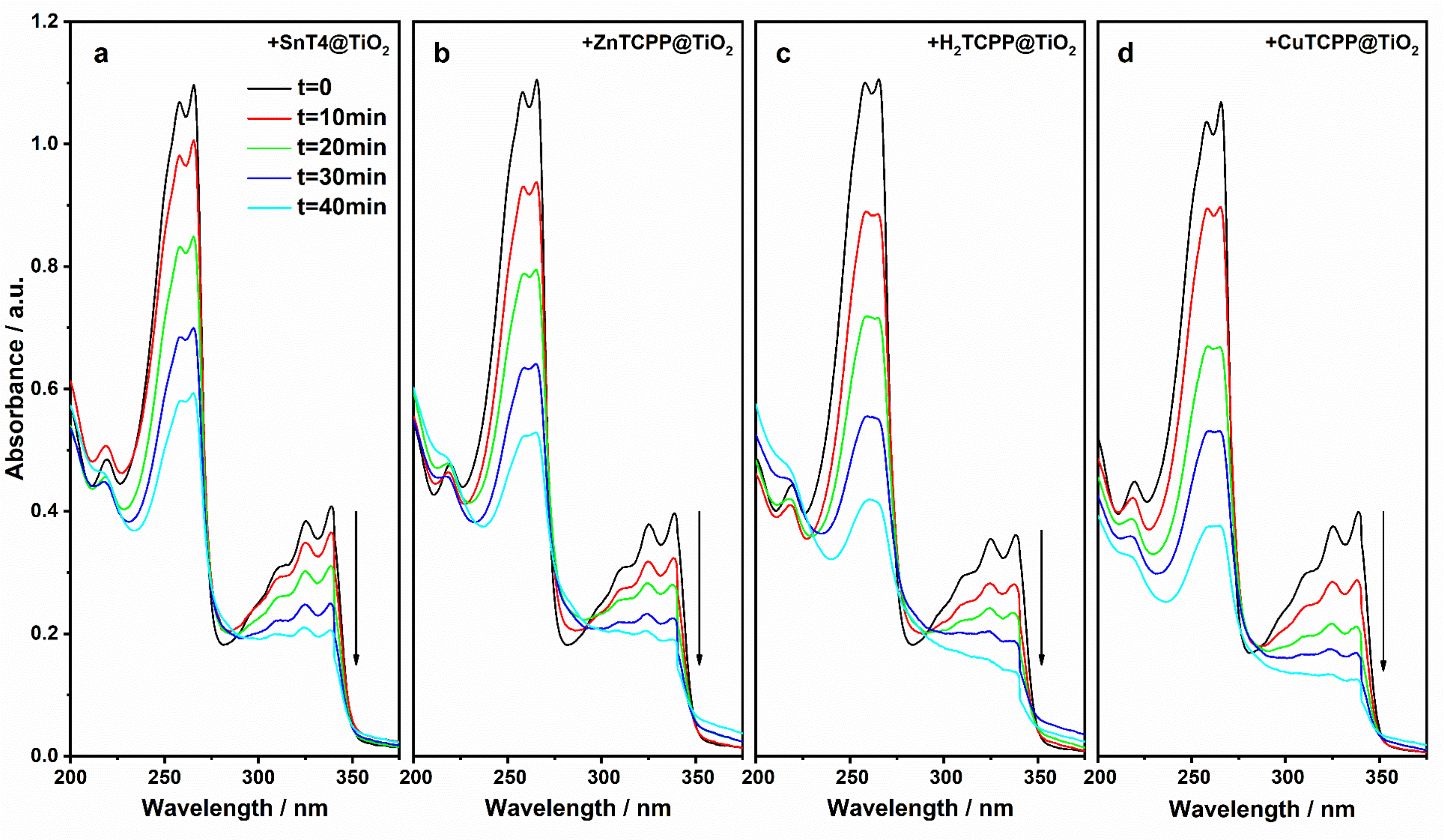

Figure S6. UV/Vis spectra (cuvette path length = 1 cm) of the photodegradation tests at different irradiation time for water solution of OXA in the presence of SnT4@TiO_2 (panel a), ZnTCPP@TiO_2 (panel b), H_2TCPP@TiO_2 (panel c) and CuTCPP@TiO_2 (panel d). In all experiments, the initial concentration of OXA was 30 μM at pH = 7.0, and the amount of photocatalyst used was 1 mg.

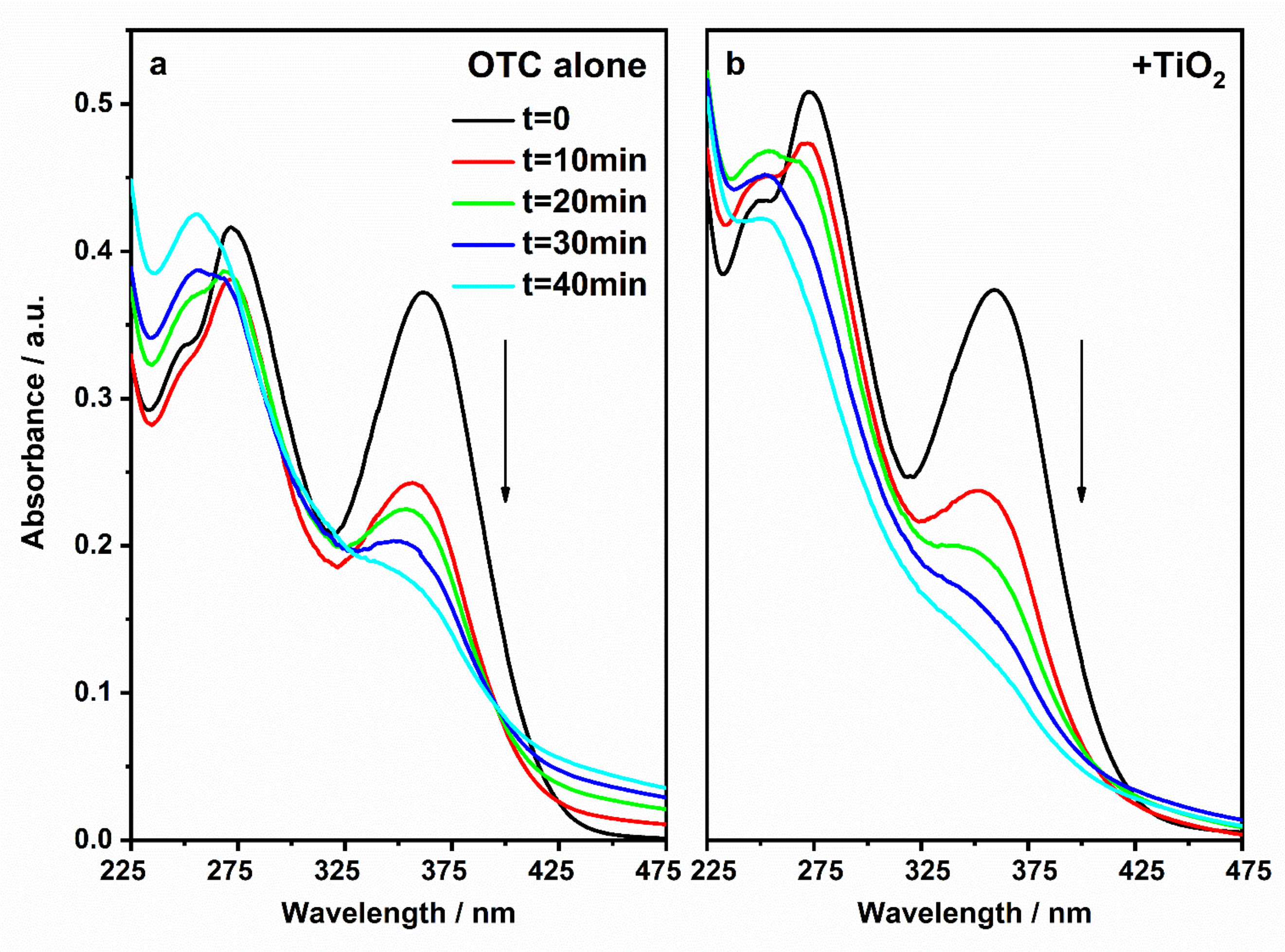

Figure S7. UV/Vis spectra (cuvette path length = 1 cm) of the photodegradation tests at different irradiation time for water solution of OTC alone (panel a) and in the presence of 1 mg of TiO_2 (panel b). In both experiments the initial concentration of OTC was 30 μM at pH = 7.0.

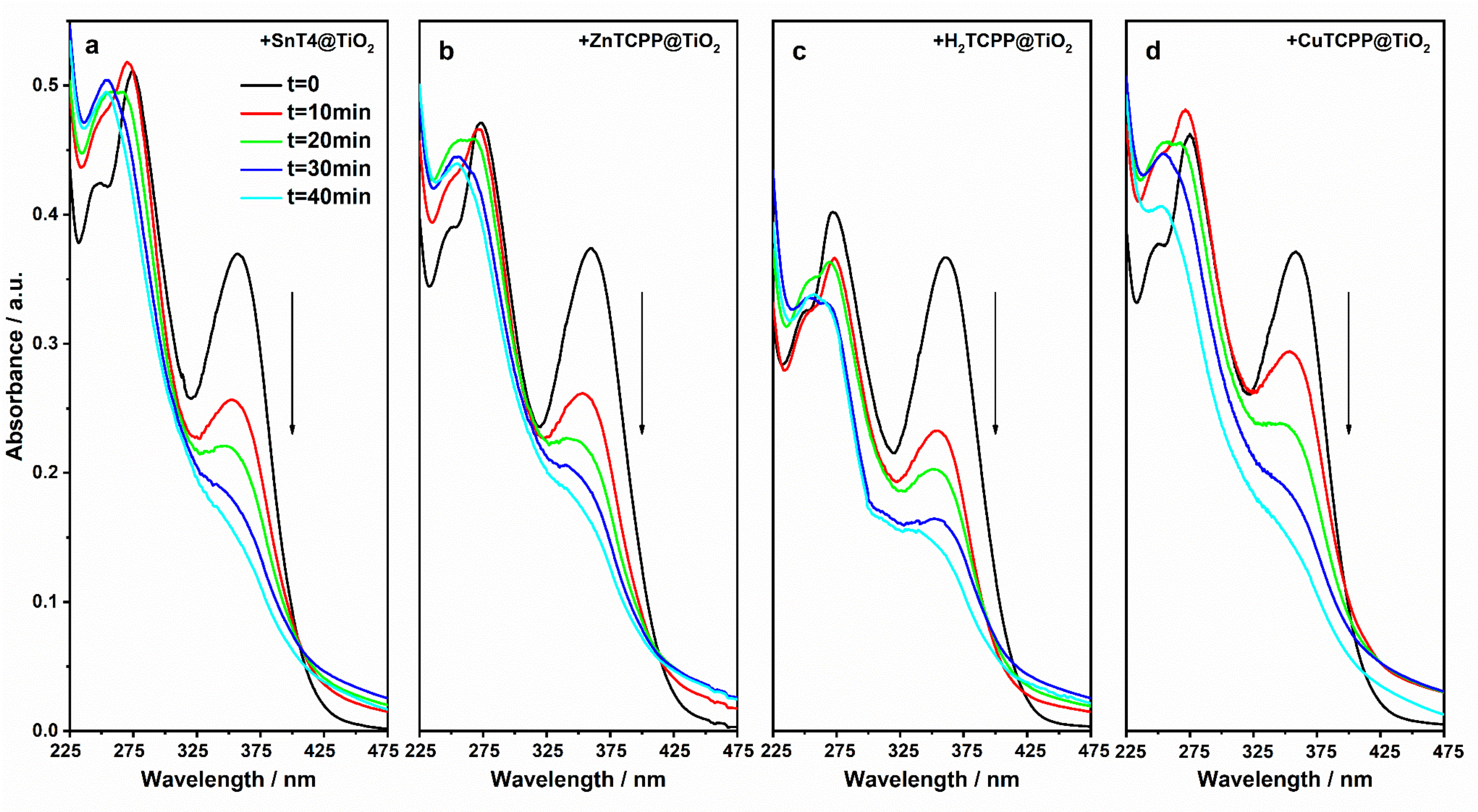

Figure S8. UV/Vis spectra (cuvette path length = 1 cm) of the photodegradation tests at different irradiation time for water solution of OTC in the presence of SnT4@TiO_2 (panel a), ZnTCPP@TiO_2 (panel b), H_2TCPP@TiO_2 (panel c) and CuTCPP@TiO_2 (panel d). In all experiments, the initial concentration of OTC was 30 μM at pH = 7.0, and the amount of photocatalyst used was 1 mg.
